# Comparison of Characteristics and DNAzyme Activity of G4–Hemin Conjugates Obtained via Two Hemin Attachment Methods

**DOI:** 10.3390/molecules23061400

**Published:** 2018-06-09

**Authors:** Joanna Kosman, Krzysztof Żukowski, Bernard Juskowiak

**Affiliations:** Laboratory of Bioanalytical Chemistry, Faculty of Chemistry, Adam Mickiewicz University, Umultowska 89b, 61-614 Poznań, Poland; krzysztof.zukowski@amu.edu.pl (K.Ż.); juskowia@amu.edu.pl (B.J.);

**Keywords:** G-quadruplex, hemin, click chemistry

## Abstract

Two conjugation methods using different linkers were applied for the investigation of the spectral characteristics and activity of G-quadruplex (G4)–hemin conjugates. For this purpose, two G-quadruplex-forming DNA sequences were selected, and then conjugated to a hemin molecule via either amine coupling or a click reaction. The products obtained via these two methods differed in their chemistry and the length of the linker between the DNA and hemin molecules. Spectral characteristics revealed that both methods produced conjugates that were more thermally stable than G4/hemin complexes. Despite similar spectral characteristics, the conjugates obtained via these two methods differed in their DNAzyme activity. G4–hemin conjugates obtained through amine coupling exhibited higher activity than conjugates obtained through a click reaction. This was potentially due to the length and chemistry of the linker, which was 30 atoms long following the click reaction, but only six atoms long following amine coupling. A longer connector favors higher flexibility, and hence, reduces the binding of hemin with G4. The aromatic groups present in the linker obtained through the click reaction can also disturb the G4–hemin interaction. However, the conjugation of G4 DNA to hemin via the click reaction was connected to a higher yield, and did not require any sophisticated synthesis equipment.

## 1. Introduction

Complexes of hemin and G-quadruplex (G4)-forming aptamers are known as DNAzymes, which exhibit horseradish peroxidase (HRP)-like activity [1,2,3]. Such HRP-mimicking DNAzymes were applied to the development of numerous biosensing devices and bioanalytical assays [2,4]. Recently, Sen et al. published a paper confirming the applicability of DNAzymes as catalysts in co-organic solvents, which extended the possibility of using these systems in industrial assays [5]. DNAzymes possess many advantages over conventional protein enzymes, including relatively inexpensive production and self-replication, stability in ambient and elevated temperatures, possibility of renaturation, and a wide range of modifications. The hemin molecule binds to the DNA oligonucleotide through an end-stacking interaction between the pyrrole rings of porphyrin and DNA nucleobases. This interaction mode requires an easy access of hemin to the G-quartet (four planar guanine residues connected via Hoogsteen hydrogen bonds); therefore, the G-quadruplexes forming parallel topologies tend to produce DNAzymes exhibiting higher peroxidase activity [6]. Outside of DNA sequence, the topology of the G-quadruplex depends on various environmental conditions, such as the presence and type of cation [7], or the addition of a molecular crowding agent [8,9]. Many attempts to enhance DNAzyme activity were undertaken using techniques such as DNA-sequence modification [10,11], or the alteration of environmental conditions [12,13]. Wang et al. studied the influence of adjacent adenine on the enzymatic activity of DNAzymes, and speculated that adenine might act similarly to distal histidines in protein peroxidases [10]. Moreover, Chang et al. proved that flanking d(CCC) sequences enhanced the peroxidase activity of DNAzymes [11]. An interesting study by Cheng et al. proved the relationship between the loop transposition of G-quadruplexes and catalytic activity [14]. In our previous paper, we demonstrated how the addition of divalent cations (Ca^2+^ and Mg^2+^) altered the G-quadruplex topology, and consequently, the catalytic activity of DNAzymes [12]. Golub et al. designed a nucleoapzyme which possessed both a substrate recognition domain (aptamer) and a reporter domain (DNAzyme) in their structure [15].

The hemin/G4 systems exhibit much lower catalytic activity than HRPs, which limits the sensitivity of DNAzyme-based assays. The main reason for the lower sensitivity of hemin/G4 systems is the dissociation of hemin from the complex (K_d_ ~10^−7^ M) [10,16]. Among the many attempts to enhance the peroxidase activity of hemin/G4 DNAzymes [2,17,18], the most promising appeared to be an approach consisting of a covalent attachment of the hemin group to the G4 oligonucleotide [19,20,21,22]. Such hemin–G4 conjugates were obtained using two synthesis routes: amine coupling [19,20,21] and a click reaction [22]. The coupling reactions of the activated ester in hemin with amino-modified G-quadruplexes were carried out as a post-synthetic step both in a bulk solution [19,21] and on a solid support [20]. Relatively low yield (~10%) is a drawback of the amine-coupling strategy for the synthesis of DNA–hemin conjugates. In our previous paper [22], we described an alternative method of hemin conjugation to a DNA oligonucleotide using the click-reaction approach, which possessed some advantages over the amine-coupling reaction, such as a higher yield, and a simple synthesis method that can be carried out without additional equipment. A brief comparison of the conjugates obtained using these two approaches indicated differences in their catalytic activity. Such differences were expected since these DNAzymes differed in the length and type of their linkers. Here, we report the spectral characteristics and catalytic-activity results for conjugates with different linkers, synthesized according to both approaches. 

## 2. Results

### 2.1. Chemistry

**Scheme 1:**
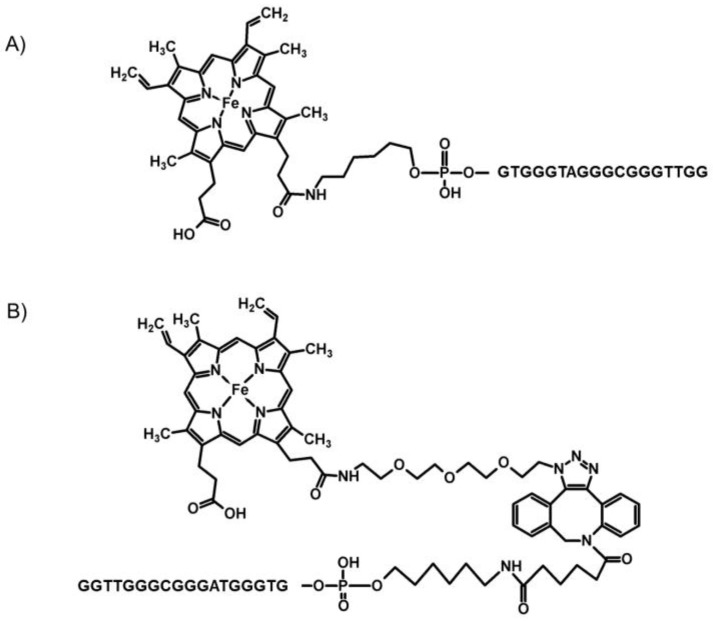
Chemical formulas of the DNA–hemin conjugates: (**A**) PS2.M–hemin obtained through the amine-coupling reaction (PS2.M–hem1), and (**B**) PS2.M–hemin obtained through the click reaction (PS2.M–hem2). Two potential isomers are possible. The scheme presents the tris isomer.

In order to investigate the effect of the conjugation method on the activity and the spectral characteristics of DNA–hemin conjugates, two sequences were selected (Table 1). Both PS2.M and CatG4 oligonucleotides form strong complexes with hemin, and the resulting DNAzymes (DNA/hemin associates) exhibit a high peroxidase activity. Conjugates possessing covalently attached hemin (DNA–hemin) were synthesized using the conventional amine-coupling reaction [20,21] (DNA–hem1), and the click reaction recently described by us [22] (DNA–hem2). The click procedure included the modification of hemin to obtain a hemin-azide derivative, with the azide group attached via a linker. Then, the hemin-azide was used in a strain-promoted alkyne-azide cycloaddition (SPAAC) reaction with a DNA oligonucleotide containing a cyclooctyne group at the 5′ end. The product of this reaction was purified using HPLC, and was identified using matrix-assisted laser deposition ionization/time of flight (MALDI-TOF) mass spectrometry (MS). The DNA–hemin conjugates synthesized using these two routes differed in the length of the linker connecting the DNA oligonucleotide and the hemin moiety. The conjugates obtained through amine coupling (route 1) possessed a six-atom-long linker, whereas conjugates obtained through the click reaction had a 30-atom-long linker. The linkers also differed in their chemistry (Scheme 1). In order to elucidate the influence of both linkers on the G-quadruplex formation and its structure, we first conducted spectral measurements for the synthesized DNA–hemin conjugates, and the reference DNA/hemin association complexes. 

### 2.2. Spectroscopic Characterization

**Figure 1:**
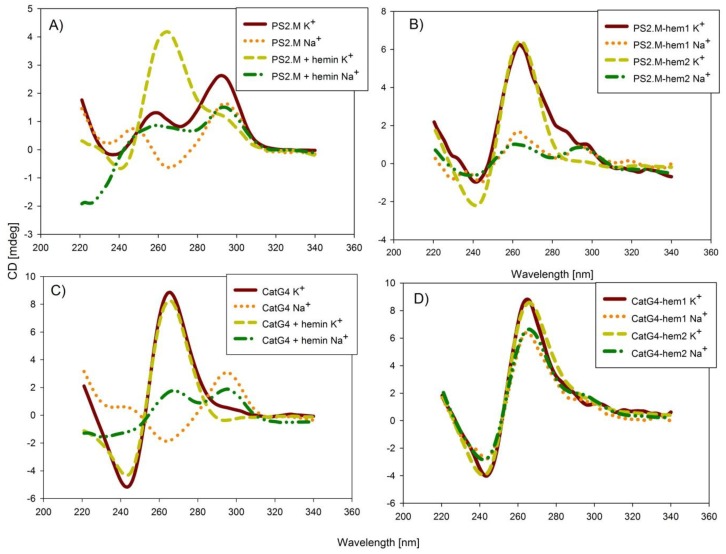
Circular dichroism (CD) spectra of the studied systems in the presence of either potassium or sodium cations: (**A**) PS2.M and PS2.M/hemin complexes, (**B**) PS2.M–hemin conjugates, (**C**) CatG4 and CatG4/hemin complexes, and (**D**) CatG4–hemin conjugates. Conditions: 2 µM DNA, 2 µM hemin (if present), 100 mM KCl or NaCl, and 10 mM Tris-HCl (pH 8.0). Experiments were performed in triplicate.

In order to determine the topology of the G-quadruplexes, we measured the circular dichroism (CD) spectra of all studied conjugates (Figure 1 and Figure S1). The circular-dichroism technique is very helpful in the determination of G-quadruplex topology, since its parallel structure gives distinctive spectra with a negative band at 240 nm and a positive band at 260 nm. In contrast, the spectra of antiparallel topology exhibit a negative band at 260 nm and a positive band at around 290 nm [23]. The PS2.M oligonucleotide in the presence of potassium cations formed a mixture of parallel and antiparallel topologies, while in the presence of sodium cations, the equilibrium shifted to the formation of mostly antiparallel species (Figure 1A). The addition of the hemin molecule to potassium-containing samples led to the stabilization of the parallel topology, with a clear increase in the band at 260 nm; however, spectral changes for the sodium-based sample suggested the formation of a mixture of both parallel and antiparallel topologies. These results support the hypothesis that the highest binding affinity of hemin is observed for parallel G-quadruplexes, and that hemin itself can force changes in G-quadruplex topology. The covalent conjugation of hemin for both PS2.M–hemin via amine coupling (PS2.M–hem1), and PS2.M–hemin via the click reaction (PS2.M–hem2) resulted in the formation of parallel G-quadruplexes in the presence of potassium cations, and mixture of parallel and antiparallel quadruplexes in the presence of sodium cations (Figure 1B). The similar spectra for the PS2.M–hemin conjugates and the PS2.M/hemin complexes suggest a similar mode of interaction exhibited by these conjugates (Figure 1A versus Figure 1B). In the case of the CatG4 association complex, the CD characteristics corresponding to parallel topology in the presence of potassium cations, and antiparallel topology in the presence of sodium cations were observed (Figure 1C). The binding of hemin resulted in the preservation of parallel topology in the presence of potassium cations, and a shift from antiparallel topology to a mixture of both topologies in the presence of sodium cations (Figure 1C). All conjugates of G-quadruplex and hemin exhibited characteristics typical for parallel topology in the presence of both sodium and potassium cations. The effect of hemin covalent conjugation on the CatG4 topology was manifested by the formation of parallel topology, even in the presence of sodium cations (Figure 1D). The higher CD signals observed for the conjugates and some association systems with hemin were probably associated with the formation of a more compact G-quadruplex structure. All CD experiments pointed to a stabilization of the parallel G-quadruplex structure due to a covalent attachment of hemin.

The next step of spectral characterization included the determination of the thermal profiles of the complexes and conjugates. The experiments were conducted for DNA oligonucleotides, DNA oligonucleotide/hemin complexes, and DNA–hemin conjugates for both studied sequences (Table 2; Figure S2). The addition of a hemin molecule resulted in the stabilization of the G-quadruplex structure, and an increase in melting temperature (T_m_). However, this effect for DNA/hemin complexes was only noticeable in the presence of potassium cations, whereas in the presence of sodium cations, the stabilization of the G-quadruplex structure due to hemin was not observed. These results agreed with the CD observations, where the addition of hemin in the presence of Na^+^ led to only small changes in the CD spectra of G-quadruplexes. Interestingly, the DNA–hemin conjugates possessed melting temperatures above 80 °C in a K^+^ environment. The melting temperatures for the conjugates in the presence of Na^+^ also increased by about 10 °C when compared with those of the DNA/hemin association systems. These results suggested that the conjugation of hemin led to the formation of more stable G-quadruplex structures. Because of the high thermal stability of the studied conjugates, the melting profiles were not fully developed in the studied temperature range (10–90 °C). To get insight into the effect of alkali-metal cations on the thermal stability of conjugates, we measured the melting profiles of the CatG4–hem1 conjugate in varying KCl and NaCl concentrations (0–100 mM) (Figures S3 and S4). The obtained T_m_ values included in Table 2 indicated that the conjugate exhibited a high melting temperature (64 ± 0.7 °C), even without the addition of any metal cations. Moreover, the presence of only 10 mM NaCl or KCl caused a substantial rise in the melting profile to higher temperatures (∆T_m_ = 9 and 21 °C, respectively). The effect of potassium was more pronounced, since 25 mM KCl shifted the melting profile further than the hampered precise T_m_ determination. 

**Figure 2:**
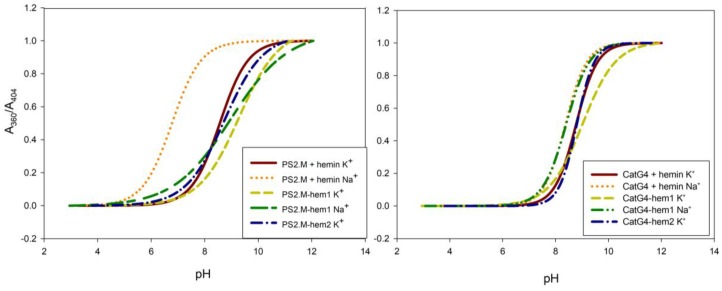
Dependences of the ratio of absorbances at 360 nm and 404 nm (A_360_/A_404_) for hemin-containing systems against the pH, allowing the determination of acid dissociation constant (pKa) values. Conditions: 1 µM DNA, 1 µM hemin (if present), and 100 mM KCl or NaCl. Experiments were performed in triplicate.

Another important characteristic of DNAzymes is the acid dissociation constant (pKa) attributed to the ionization of a water molecule coordinated to the axial position of the hemin Fe(III) atom. From the literature data and our own experience, we know that this value is correlated with the activity level of DNAzymes [24]. In order to determine the pKa of the studied compounds, the UV-VIS absorption spectra of DNA/hemin complexes and of DNA–hemin conjugates were measured at various pH values. Based on the ratio of absorbance at 360 nm to that at 404 nm, the dependences of absorbance ratio on pH were plotted (Figure 2 and Figure S5). The curves in Figure 2 possess a sigmoidal shape, and it was possible to determine pKa values of hemin in the investigated systems using a first-derivative approach. The values of pKa for all studied systems are gathered in Table 3. The systems in the K^+^ environment exhibited a pKa around 9, which agreed with their expectedly high peroxidase activity. In the presence of Na^+^, the PS2.M/hemin complex showed a significantly lower pKa (about 2 units). In contrast, the pKa for G4–hemin conjugates exhibited similar values in the presence of both sodium and potassium ions. This can be clearly seen in the cases of DNAzymes based on the CatG4 sequence, for which the differences in pKa for K^+^ systems versus Na^+^ systems were negligible. All these results pointed to a higher efficiency of DNA–hemin conjugates, which was not dependent on the presence of potassium or sodium cations. 

### 2.3. Activity of DNAzymes

**Figure 3:**
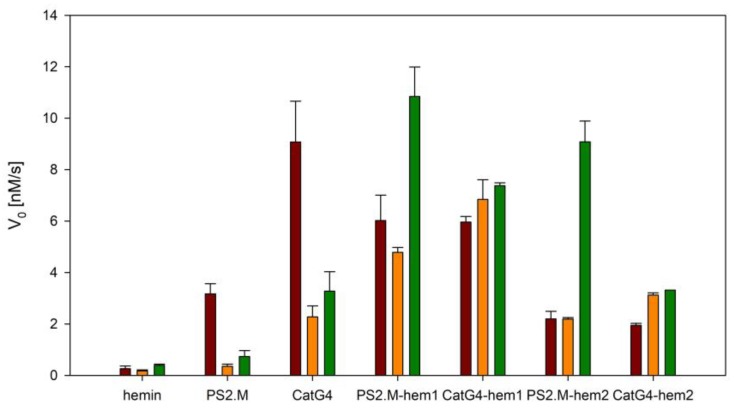
Catalytic activity of various enzymatic systems in an oxidation reaction of 2,2′-azino-bis(3-ethylbenzothiazoline-6-sulphonic acid) (ABTS) with hydrogen peroxide. The colors of the bars represent the reactions in the presence of potassium cations (brown) and sodium cations (orange), or in the absence of cations (green). Conditions: 0.5 µM DNA, 0.5 µM hemin (if present), 100 mM KCl or NaCl, 10 mM Tris-HCl (pH 8.0), 1 mM ABTS, and 1 mM H_2_O_2_. The values were averaged across three experiments.

To verify the peroxidase activity of studied DNAzyme complexes and conjugates, their catalytic efficiencies were tested in an oxidation reaction of 2,2′-azino-bis(3-ethylbenzothiazoline-6-sulphonic acid) (ABTS) with hydrogen peroxide. ABTS is a popular chromogenic substrate for HRP, and is frequently used in various bioassays [25,26]. The results of the initial reaction rates for all systems are shown as a bar plot in Figure 3, and catalytic profiles are presented in Figure S6. The DNAzyme based on the PS2.M/hemin complex exhibited good activity in the presence of potassium cations, and almost no activity in either the presence of sodium cations or the absence of cations. The covalent attachment of hemin using amine coupling led to an increase in activity for all studied cases, with a clear superiority for the PS2.M–hem1 DNAzyme in the absence of any metal cations. A similar situation was observed for the PS2.M–hem2 conjugate synthesized using the click reaction. In the case of DNAzymes based on the CatG4 sequence, the activities of both conjugates were lower than that of the CatG4/hemin complex in the presence of K^+^. It can be seen that conjugates synthesized using the amine-coupling reaction (CatG4–hem1) possessed higher activity than CatG4–hem2, which was obtained through the click reaction. 

**Figure 4:**
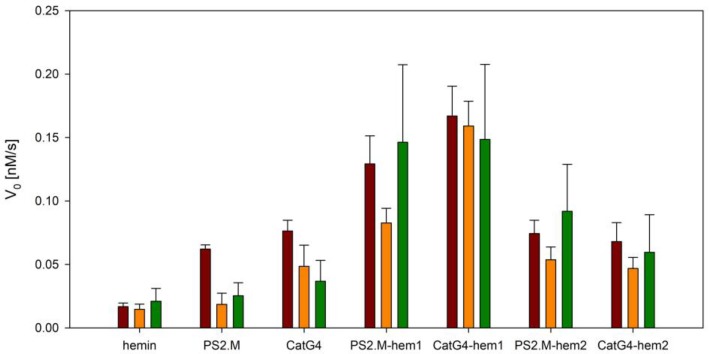
Peroxidase activity of the studied systems in the oxidation of Amplex Red. The colors of the bars represent various conditions: the presence of potassium cations (brown) and sodium cations (orange), or the absence of cations (green). Conditions: 0.05 µM DNA, 0.05 µM hemin (if present), 100 mM KCl or NaCl, 10 mM Tris-HCl (pH 8.0), 1 µM Amplex Red, and 10 µM H_2_O_2_. The values were averaged across three experiments.

An explanation of the observed variation in catalytic activity of the tested systems may involve the selectivity of DNAzymes toward particular substrates [27]. In order to verify whether the activity of DNAzymes varied upon changing the substrate, we examined the oxidation reaction of a fluorogenic substrate, Amplex Red, using associated and conjugated DNAzymes. Figure 4 shows the initial rates for the studied systems for the oxidation reaction of Amplex Red with fluorescent resorufin (catalytic profiles are presented in Figure S7). Using this substrate, the conjugates synthesized using the amine-coupling approach also exhibited higher activity than those synthesized through the click reaction. However, in this case, the activity of CatG4–hem1 was higher than that of the CatG4/hemin complex. Interestingly, the same behavior was not observed for CatG4–hem2. Unlike the PS2.M conjugates, which showed the highest activity in the absence of metal cations for both substrates, the CatG4 conjugate DNAzymes catalyzed the oxidation of Amplex Red with comparable efficiency in both the absence and presence of potassium ions. In conclusion, we observed that the presence of metal cations was not a crucial factor limiting the peroxidase activity of G4–hemin conjugates. This finding opens up novel areas for the potential applications of DNAzymes. 

**Figure 5:**
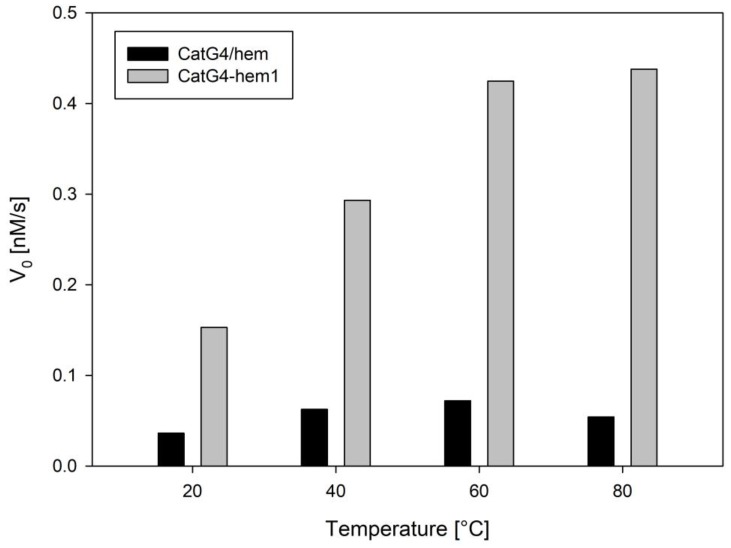
Peroxidase activity of the CatG4/hemin complex (black) and the CatG4–hem1 conjugate (gray) at various temperatures. Conditions: 0.05 µM DNA, 0.05 µM hemin (if present), 100 mM KCl, 10 mM Tris-HCl (pH 8.0), 1 µM Amplex Red, and 10 µM H_2_O_2_.

Recently, Gao et al. published a paper concerning DNAzyme activity in elevated temperatures [28]. Since studied conjugates showed higher thermal stability in comparison with DNA/hemin complexes, we examined their catalytic activities at various temperatures (Figure 5). Experiments were performed using the CatG4/hemin association complex and the CatG4–hem1 conjugate as representative systems. The results in Figure 5 indicated that the activity of the conjugate doubled at 40 °C, and increased even further at 80 °C. In contrast, for the CatG4/hemin complex, only small increases were observed at 40 °C and 60 °C; however, the activity decreased at 80 °C. It is generally well known that an increase in the temperature of catalytic reactions causes an increase in reaction rates, and this trend was observed for both systems in the lower temperature range. However, higher temperatures may influence the stability of DNAzymes (melting), which disrupts the G-quadruplex structure, and hence, its activity. These two opposing phenomena contribute to the overall activity of DNAzymes. The results shown in Figure 5 indicated that, at higher temperatures, the associate DNAzyme was denatured (unfolding of the G-quadruplex structure), while the conjugate preserved its structure, and thus, its activity. Therefore, DNA–hemin conjugates are a better alternative for the application of biocatalysis at higher temperatures.

## 3. Discussion

The spectroscopic characterization of the studied systems revealed that a conjugation of hemin to a G-quadruplex-forming oligonucleotide enforced the formation of parallel G-quadruplexes for PS2.M conjugates, in the presence of potassium cations. In the presence of sodium cations, a mixture of parallel and antiparallel G-quadruplexes was formed. For CatG4 conjugates, parallel G-quadruplexes were formed, even in the presence of sodium cations. These observations proved that the covalent attachment of hemin to oligonucleotides influenced the topology of G-quadruplexes, enforcing a transformation to a structure that facilitated efficient hemin binding, and possessed higher peroxidase-like activity. The conjugation of hemin also influenced the thermal stability of the G-quadruplex structure. In all cases, hemin conjugation resulted in an increase in melting temperature (>80 °C) in the presence of potassium cations. The stability of conjugates in the presence of Na^+^ also increased by 10–40 °C. The high stability of the DNA–hemin G-quadruplex is an advantageous feature, since it allows for a broader application of DNAzymes. The spectroscopic characterization also revealed that the systems synthesized through amine coupling and the click reaction exhibited similar spectral properties. Both systems possessed high comparable pKa values (around 9), which was proven to be a crucial condition for the high activity of DNAzymes.

Despite rather small differences in the CD spectra, melting temperatures, and pKa values, the catalytic activity significantly varied for conjugates synthesized via different approaches. Generally, conjugates synthesized through amine coupling exhibited higher activity when compared to those obtained through the click reaction. The same dependence was observed regardless of the nature of substrate used for the peroxidase reaction. Since both conjugates were based on the same sequences, the only difference that could influence the catalytic efficiency of the conjugate should be ascribed to the type of linker. As mentioned above, the linkers differed in length and chemistry. The length of the linker in the conjugates obtained through the click reaction (30 atoms) possessed higher flexibility, which may result in the conformational freedom of the hemin molecule, and an imperfect interaction with the G-quadruplex. In the case of conjugates obtained through the amine-coupling reaction, the linker was very short (6 atoms), resulting in its restricted flexibility; however, it was sufficiently long to ensure better binding of hemin to the G-quadruplex, which resulted in higher activity of these conjugates. Additionally, the hydrophobic aromatic fragment present in the linker of conjugates obtained through the click reaction may interrupt the proper binding of hemin to the G-quadruplex. From the presented results, it was not possible to conclude whether the hemin molecule bound to the 3′ or 5′ G-quadruplex. This issue requires more research on the mechanism of hemin binding to G-quadruplex, including syntheses of other conjugates which also contain a hemin linked to the 3′ end.

## 4. Materials and Methods 

### 4.1. Materials

The hemin-modified DNA oligonucleotides (active-ester route) were synthesized through an amine-coupling reaction by Future Synthesis (Poland) according to a procedure published by Niemeyer [18]. The products were of HPLC purity, and were used without any further purification. Hemin–G4 DNA conjugates were synthesized using SPAAC (strain-promoted alkyne-azide cycloaddition) click chemistry, according to the procedure previously developed in our laboratory [23]. The products were purified using HPLC, and characterized using MALDI-TOF MS. The oligonucleotides possessing the cyclooctyne group were purchased from Metabion (Germany), and the unmodified oligonucleotides were purchased from Genomed (Poland). The sequences of all studied oligonucleotides are shown in Table 1. The concentrations of all oligonucleotide samples were quantified using UV-VIS absorption spectroscopy at 85 °C with the following extinction coefficients for nucleobases at 260 nm (M^−1^ cm^−1^): A = 15,400, T = 8700, G = 11,500, and C = 7400 [29]. Hemin, ABTS (2,2′-azinobis(3-ethylbenzothiozoline)-6-sulfonic acid), Amplex Red, Brij58, and Triton X-100 were purchased from Sigma-Aldrich. A stock solution of hemin was prepared in DMSO at a concentration of 0.01 M, and was stored in a freezer for up to one month. The other reagents were of analytical grade, and were used as received. 

### 4.2. Circular Dichroism and Thermal Denaturation Experiments

CD spectra measurements were performed on a J-1500 spectropolarimeter (Jasco, Tokyo, Japan) in quartz cuvettes with a path length of 1 cm, and were averaged across three scans. CD spectra were recorded with a 100 nm/min scanning speed, and a bandwidth of 1 nm. All samples contained 2 µM oligonucleotide, 10 mM Tris-HCl buffer (pH 8.0), and required cations (if present) at a 100 mM concentration. The samples were heated at 90 °C for 5 min prior to the experiment, and allowed to cool on ice for 15 min. Melting experiments were conducted using a 1 °C/min heating/cooling rate in the 10–90 °C temperature range. All experiments were performed in quartz cuvettes with a path length of 1 cm.

### 4.3. Spectrophotometric Determination of Hemin pKa

The values of pKa were determined for hemin/DNA complexes and conjugates by analyzing the UV-VIS absorption spectra recorded at various pH values (4–11). Prior to the experiments, the samples containing 1 µM DNA/hemin complex or DNA–hemin conjugate, 10 mM Tris-HCl, and 100 mM KCl (or NaCl) were heated for 5 min at 90 °C, and then cooled on ice for 15 min. The pH was adjusted to the required value using 1 M NaOH or 1 M HCl, while the pH was controlled directly in the cuvette with a combined glass microelectrode. UV-VIS spectra (200–600 nm) were recorded after subsequent pH changes (addition of acid/base). All UV-VIS experiments were performed using a Cary 100 spectrophotometer (Agilent Technologies, Santa Clara, United States).

### 4.4. Peroxidase Activity Measurements

The samples containing 0.5 µM DNA, 0.5 µM hemin (if present), 10 mM Tris-HCl buffer (pH 8.0), and required cations were heated for 5 min at 90 °C, and allowed to cool on ice for 15 min. After addition of the peroxidase substrate (ABTS or Amplex Red), samples were transferred into microplate wells. The time profiles of absorbance or fluorescence were recorded using a M200 microplate reader (Tecan, Mannedorf, Switzerland), after initialization of the reaction with the addition of H_2_O_2_. Absorbance changes for ABTS oxidation were monitored at 414 nm, while fluorescence for the Amplex Red oxidation product (resorufin) was excited at 570 nm, and observed at 585 nm.

## 5. Conclusions

The conjugates obtained via both methods exhibited similar spectroscopic characteristics. The covalent conjugation of hemin caused a shift in G-quadruplex topology toward a parallel structure, as well as the stabilization of the G-quadruplex structure (T_m_ > 80 °C). However, the activity experiments revealed that conjugates obtained via different methods differed in their catalytic activity. The differences in activity were as a result of the linker’s length and chemistry. The longer linker, with an additional aromatic group, in conjugates obtained through the click reaction influenced a weaker interaction of hemin with the G-quadruplex when compared with that of conjugates obtained through amine coupling. Definite proof for the importance of the length and chemistry of the linker connecting hemin and the oligonucleotide in the conjugates can be provided through the design and click-reaction synthesis of a new conjugate containing a shorter linker (shorter amino–azide connector). The study on novel conjugates will be continued accordingly, since the click reaction gave a better yield, was easier to perform, and did not require sophisticated equipment. In many cases, these advantages are more important than a slightly higher activity level.

## Figures and Tables

**Table 1:** Sequences of the oligonucleotides and conjugates used in this study.

Name	Sequence
PS2.M	5′–GTG GGT AGG GCG GGT TGG–3′
CatG4	5′–TGG GTA GGG CGG GTT GGG AAA–3′
PS2.M–hem1	Hemin–(C_6_H_12_)–5′–GTG GGT AGG GCG GGT TGG–3′
PS2.M–hem2	Hemin–(C_35_N_5_O_5_H_49_)–5′–GTG GGT AGG GCG GGT TGG–3′
CatG4–hem1	Hemin–(C_6_H_12_)–5′–TGG GTA GGG CGG GTT GGG AAA–3′
CatG4–hem2	Hemin–(C_35_N_5_O_5_H_49_)–5′–TGG GTA GGG CGG GTT GGG AAA–3′

**Table 2:** Melting temperatures (T_m_) for DNA oligonucleotides and conjugates following circular dichroism (CD) spectroscopy at 260 nm. The values were averaged across three experiments.

Sequence	T_m_ [°C], 100 mM KCl	T_m_ [°C], 100 mM NaCl
PS2.M	56.5 ± 0.9	46.0 ± 0.4
PS2.M/hemin	64.6 ± 1.0	45.2 ± 0.6
PS2.M–hem1	>80.0 ^a^	>80.0 ^a^
PS2.M–hem2	>80.0 ^a^	58.4 ± 1.0
CatG4	79.2 ± 1.5	41.8 ± 0.5
CatG4/hemin	>80.0 ^a^	42.2 ± 1.4
CatG4–hem2	>80.0 ^a^	53.0 ± 1.9
CatG4–hem1	>80.0 ^a^	>80.0 ^a^
	>80.0 ^a,b^	>80.0 ^a,b^
	>80.0 ^a,c^	79.6 ± 0.3 ^c^
	85.2 ± 1.2 ^d^	73.0 ± 0.4 ^d^
	64.0 ± 0.7 ^e^	64.0 ± 0.7 ^e^

^a^ exact value impossible to be determined due to incomplete melting profile; salt concentration: **^b^** 50 mM, **^c^** 25 mM, **^d^** 10 mM, **^e^** no salt. DNA/hemin refers to the associated complex. DNA–hem1 refers to the conjugate obtained through amine coupling. DNA–hem2 refers to the conjugate obtained through the click reaction.

**Table 3:** Values of the acid dissociation constant (pKa) for the studied systems determined in the presence of potassium or sodium cations. The pKa values were averaged across three experiments.

Sequence	K^+^	Na^+^
PS2.M/hemin	>9 ^1^	6.9 ± 0.2
PS2.M–hem1	9.2 ± 0.1	9.1 ± 0.2
PS2.M–hem2	8.7 ± 0.1	Nd
CatG4/hemin	>9 ^1^	8.5 ± 0.2
CatG4–hem1	8.8 ± 0.2	8.6 ± 0.3
CatG4–hem2	8.7 ± 0.3	Nd

^1^—exact value impossible to be determined due to an incomplete profile. nd—no data.

## Supplementary Materials

The following are available online, Figure S1: CD spectra of studied systems without cation presence, Figure S2: Melting profiles of studied systems, Figure S3: Melting profile of CatG4-hem1 in various KCl concentration, Figure S4: Melting profile of CatG4-hem1 in various NaCl concentration, Figure S5: Determination pf pKa values. Plots of A360/A440 ratio against pH for studied systems, Figure S6: Catalytic profiles of ABTS oxidation in various cationic conditions: A) without cations, B) 100 mM NaCl, C) 100 mM KCl, Figure S7: Catalytic profiles Amplex Red oxidation to resorufine in various cationic conditions: A) without cations, B) 100 mM NaCl, C) 100 mM KCl.
