# Supplementary material for: Comparison of Characteristics and DNAzyme Activity of G4–Hemin Conjugates Obtained via Two Hemin Attachment Methods
Source: Molecules. 2018 Jun 9;23(6):1400. doi: 10.3390/molecules23061400 (PMC6100319; doi:10.3390/molecules23061400)
Supplement: Supplementary file 1 [file molecules-23-01400-s001.pdf]

Comparison of Characteristics and DNAzyme Activity of G4–Hemin Conjugates Obtained via Two Hemin Attachment Methods

Joanna Kosman *, Krzysztof Żukowski and Bernard Juskowiak

Laboratory of Bioanalytical Chemistry, Faculty of Chemistry, Adam Mickiewicz University, Umultowska 89b, 61-614 Poznań, Poland; krzysztof.zukowski@amu.edu.pl (K.Ż.); juskowia@amu.edu.pl (B.J.);

* Correspondence: kosman@amu.edu.pl; Tel.: +48-618-291-768; Fax: +48-618-291-555

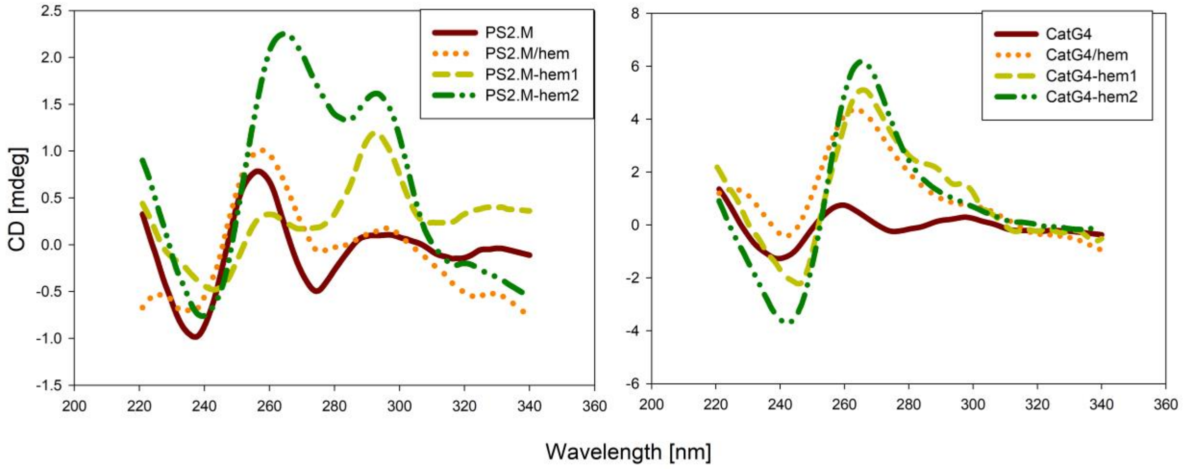

Figure S1. CD spectra of studied systems without cation presence.

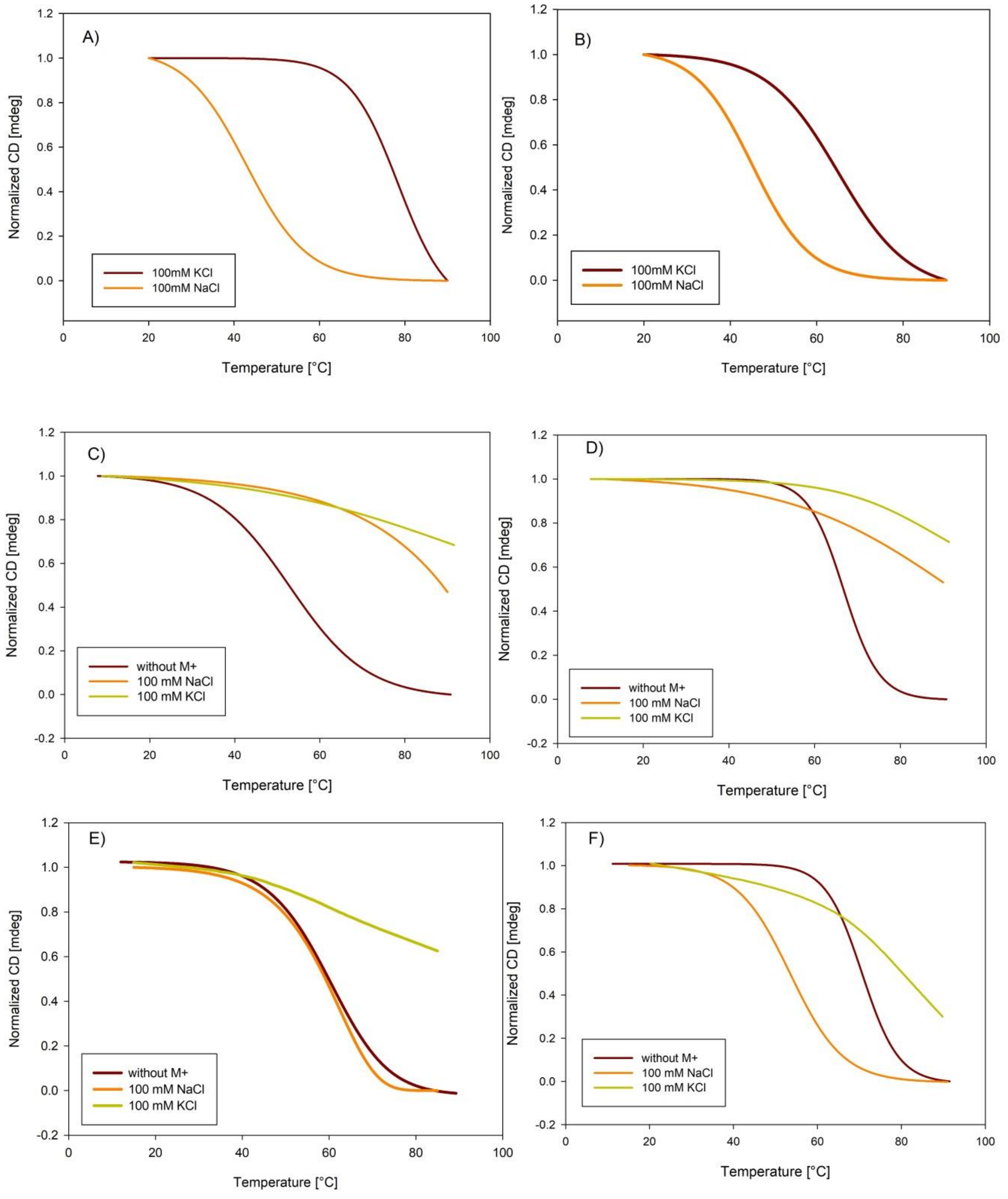

Figure S2. Melting profiles of studied systems: A) PS2.M/hem complex, B) CatG4/hem complex, C) PS2.M-hem1 conjugate, D) CatG4-hem1 conjugate, E) PS2.M-hem2 conjugate, D) CatG4-hem2 conjugate).

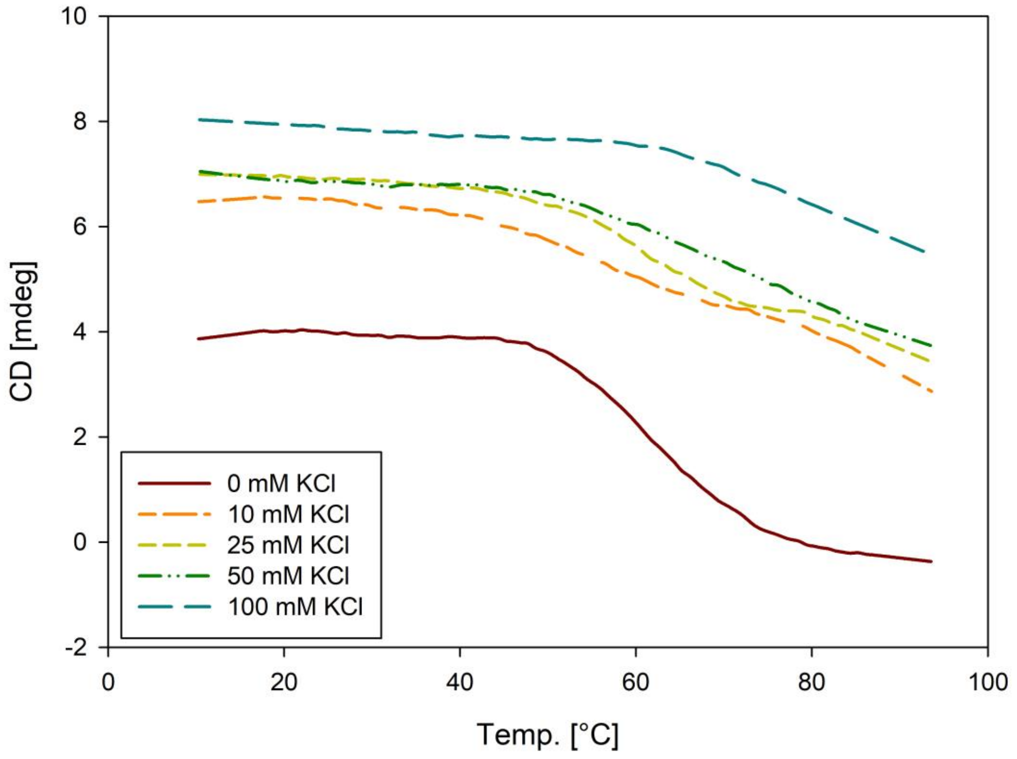

Figure S3. Melting profile of CatG4-hem1 in various KCl concentration.

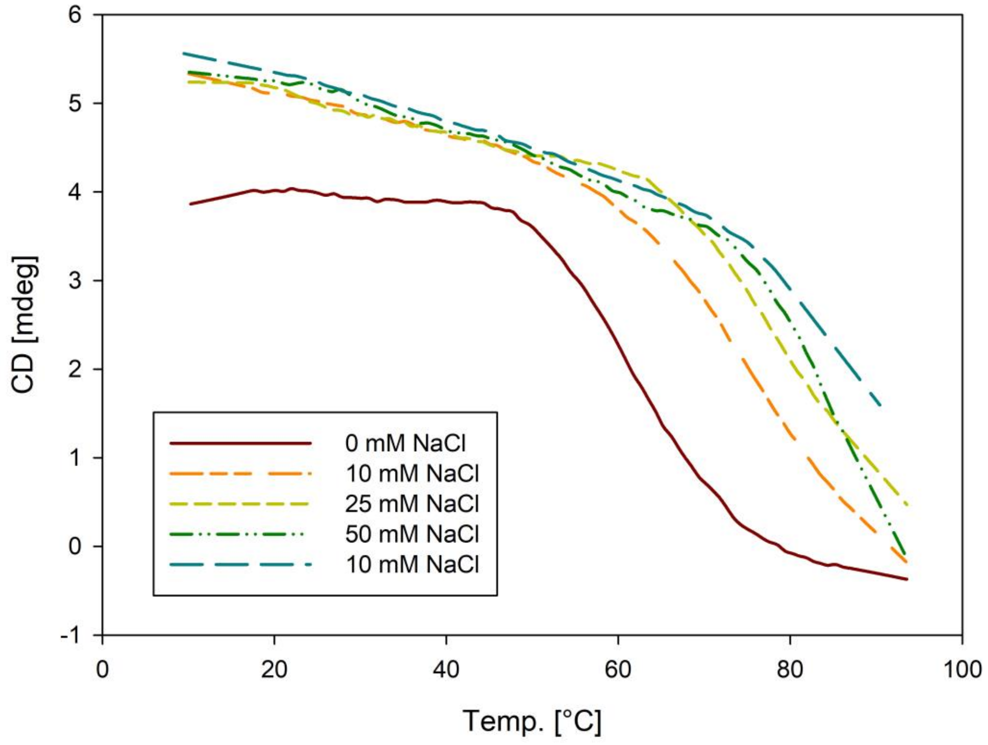

Figure S4. Melting profile of CatG4-hem1 in various NaCl concentration.

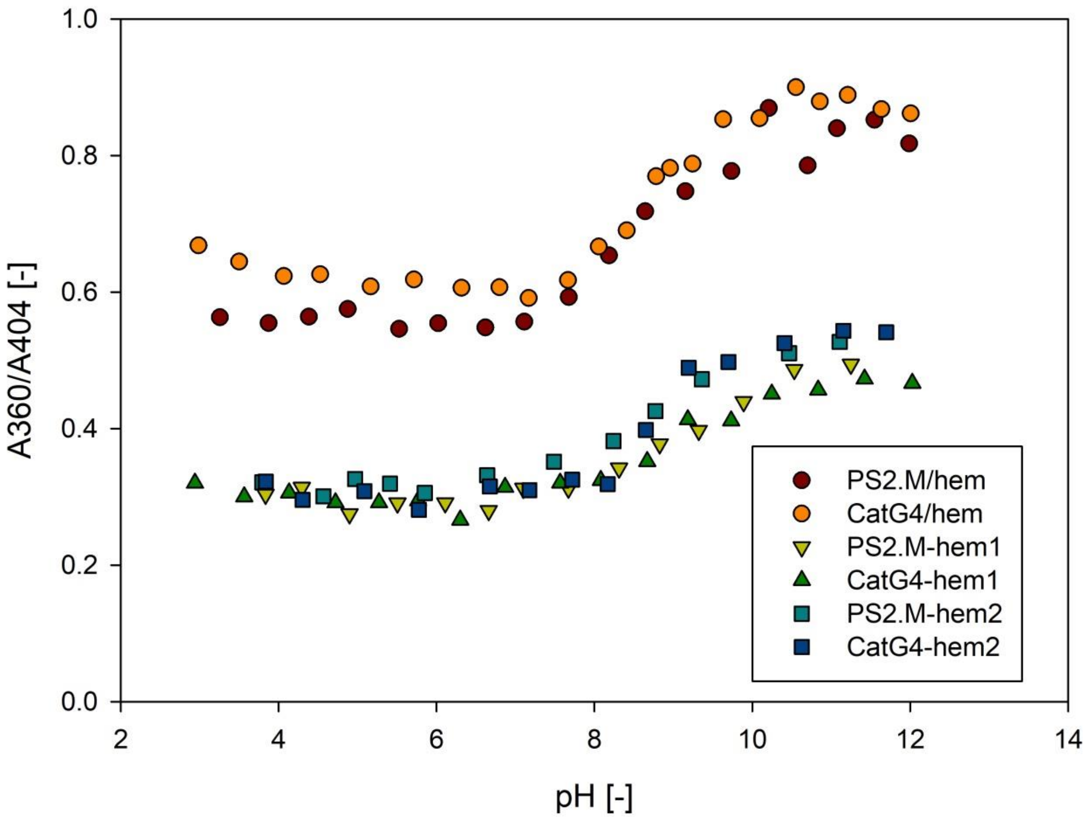

Figure S5. Determination of pKa values. Plots of A360/A404 ratio against pH for studied systems.

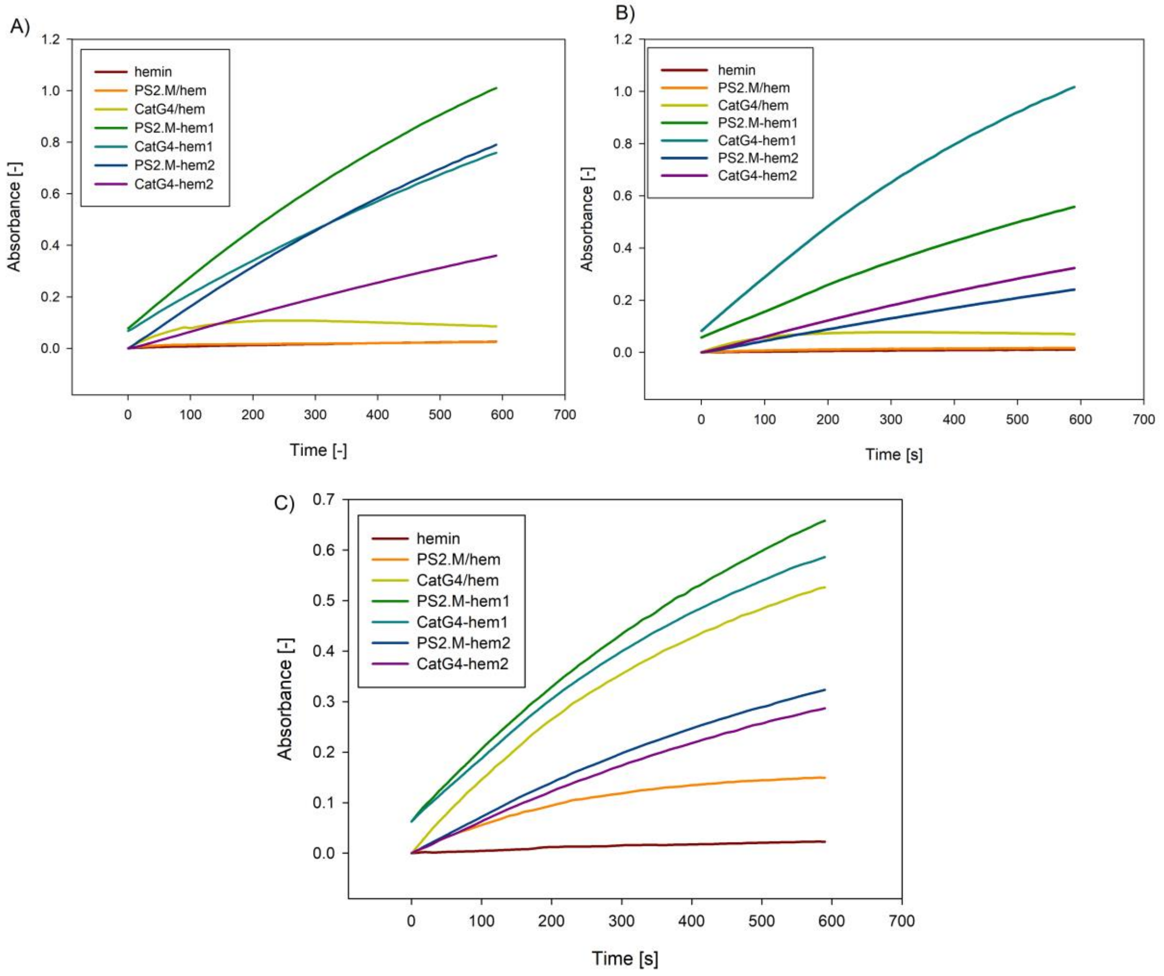

Figure S6. Catalytic profiles of ABTS oxidation in various cationic conditions: A) without cations, B) 100 mM NaCl, C) 100 mM KCl.

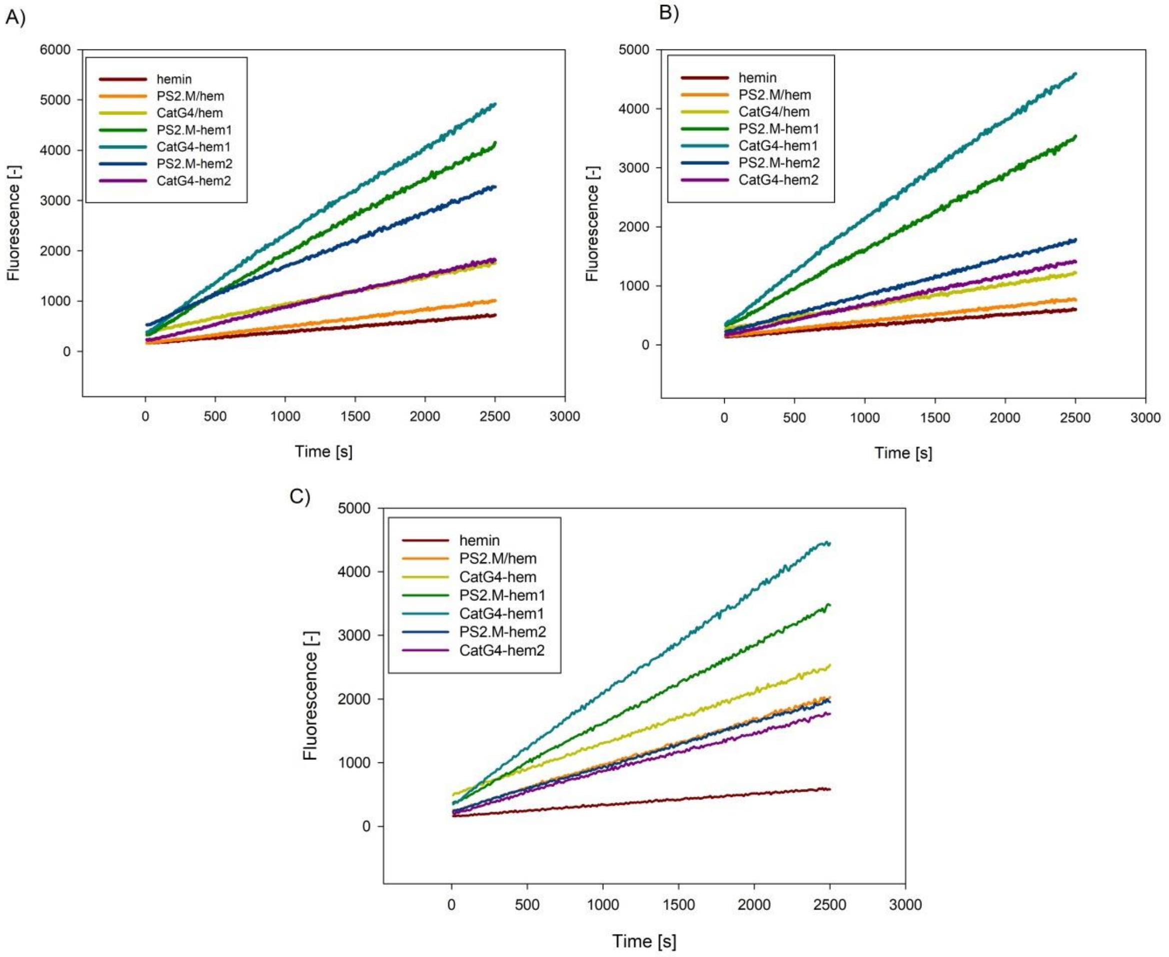

Figure S7. Catalytic profiles Amplex Red oxidation to resorufine in various cationic conditions: A) without cations, B) 100 mM NaCl, C) 100 mM KCl.
